# FOXM1-AXL/eEF2K Targeting: A Promising Treatment Strategy for Glioblastoma Multiforme Tumors

**DOI:** 10.3390/cancers17162662

**Published:** 2025-08-15

**Authors:** Christina Piperi, Kostas A. Papavassiliou, Athanasios G. Papavassiliou

**Affiliations:** 1 Department of Biological Chemistry, Medical School, National and Kapodistrian University of Athens, 11527 Athens, Greece; 2 First University Department of Respiratory Medicine, ‘Sotiria’ Chest Hospital, Medical School, National and Kapodistrian University of Athens, 11527 Athens, Greece; konpapav@med.uoa.gr

In a recent study, Biltekin and colleagues [1] demonstrated that THE inhibition of a master transcription factor, forkhead-box protein M1 (FOXM1, a proliferation-associated transcription factor that is broadly spatiotemporally expressed during the cell cycle and is closely engaged in tumorigenesis), in glioblastoma multiforme (GBM) cells can lead to the suppression of vital cell functions through the regulation of the AXL/eukaryotic elongation factor 2 kinase (eEF2K) signaling axis, inducing apoptosis and ferroptosis. The authors have initially employed data mining to detect the enrichment of eEF2K, AXL (also known as ARK, Tyro7, or JTK11), and FOXM1 in GBM tumors compared TO non-malignant brain tissue, further validating their overexpression in several GBM cell lines. By transfecting different GBM cell lines with specific small interfering RNAs (siRNAs) for each protein, they first showed the reduced cell proliferation and clonogenic potential of GBM cells, followed by the verification of the direct physical interaction of the three proteins. Subsequently, knockdown of *FOXM1*, *AXL*, and *eEF2K* suppressed spheroid formation, as well as cell migration and invasion, in GBM cells. Importantly, downregulation of these three proteins triggered apoptosis and ferroptosis in GBM cells and enhanced temozolomide (TMZ)-induced cell death, highlighting the beneficial effect of this combinatorial targeting in overcoming TMZ resistance [1].

This mechanistic study has several significant therapeutic insights that are worth discussing. The foremost of these is the dual-targeting potential of FOXM1 inhibition, which is commonly overexpressed in GBM [2], in inducing the downregulation of both AXL—a receptor tyrosine kinase (RTK) linked to therapy resistance and mesenchymal phenotype in GBM [3]—and eEF2K, which promotes cell survival via pro-growth translational control. Notably, this combined suppression potentiates both apoptosis and ferroptosis in GBM cells, bypassing resistance mechanisms [4]. Therefore, by priming GBM cells to ferroptosis, FOXM1 inhibition offers a compelling strategy to target treatment-resistant subpopulations.

In addition, various clinical AXL inhibitors have been designed and are presently being investigated in solid tumors [5]. Some of them (e.g., the small-molecule ATP-competitive inhibitor BMS-777607) have already been tested in preclinical GBM models, exhibiting reduced growth, migration, and invasiveness while inducing apoptosis and halting angiogenesis [6]. Accordingly, a synergy of FOXM1 inhibition and AXL blockade may be favorable in impeding both cell signaling and mechanisms of survival.

Moreover, eEF2K, although less widely studied, has been shown to support tumor survival by modulating translational control and resistance to stress [7]. Genetic or pharmacological inhibition of eEF2K fosters apoptosis and autophagy in a spectrum of tumor types, including gliomas [8]. Therefore, the combined inhibition of FOXM1 and eEF2K may induce cell death and minimize translational-driven resistance. To this end, a significant biomarker potential has also been revealed, since measuring FOXM1/AXL/eEF2K levels could identify the patients most likely to respond to treatment.

Overall, the study by Biltekin et al. suggests a central role for FOXM1 in GBM survival, and provides a promising precision-oriented therapeutic approach which, by employing FOXM1 inhibitors to downregulate oncogenic signaling, enables AXL inhibition to hinder invasion and survival and eEF2K blockading to reinforce cell death signaling, eventually activating ferroptosis to execute cell death. Efforts to encapsulate FOXM1 inhibitors (with AXL and/or eEF2K blockers) in blood–brain barrier (BBB)-permeant formulations guided by biomarker monitoring may prove to be highly effective against aggressive GBM tumors that are resistant to current regimens. Although a careful safety profiling is needed, this multi-modal approach holds great promise for brain tumor therapy in the foreseeable future.
